# Potential Application of Eicosapentaenoic Acid Monoacylglyceride in the Management of Colorectal Cancer

**DOI:** 10.3390/md15090283

**Published:** 2017-09-04

**Authors:** Caroline Morin, Enrique Rodríguez, Pierre U. Blier, Samuel Fortin

**Affiliations:** 1 SCF Pharma, Ste-Luce, QC G0K 1P0, Canada; cmorin@scfpharma.com; 2 Department of Biology, Université du Québec à Rimouski, Rimouski, QC G5L 3A1, Canada; Enrique_Rodriguez@uqar.ca (E.R.); Pierre_Blier@uqar.ca (P.U.B.)

**Keywords:** EPA, colorectal carcinoma, apoptosis, VEGF, EGFR, HIF1α

## Abstract

Background: There is increasing evidence that marine omega-3 oils are involved in the reduction of cancer risk and progression. However, the anticancer effect of omega-3 monoglyceride on colorectal cancer has yet to be assessed. The goal of this study was to evaluate the anti-cancer effects of eicosapentaenoic acid monoglyceride (MAG-EPA) in HCT116 colorectal carcinoma cells. Methods: The effect of MAG-EPA was evaluated in vitro on HCT116 cells and in vivo on mouse model of HCT116 xenograft. Results: Our data reveal that MAG-EPA decreased cell proliferation and induced apoptosis in HCT116 cells. In a xenograft mouse model, daily *per os* administration of MAG-EPA reduced tumor growth. Furthermore, MAG-EPA treatments decreased EGFR, VEGFR, and AKT activation pathways and reduced VEGF and HIF1α expression levels in tumors. Conclusion: MAG-EPA may promote apoptosis and inhibit growth of tumors by suppressing EGFR and VEGFR activation pathways. Altogether, these data provide new evidence regarding the mode of action of MAG-EPA in colorectal cancer cells.

## 1. Introduction

At the present time, approximately 60% of approved cancer treatment drugs are of natural origin [1]. An estimated 14,000 pharmacologically active compounds have been isolated from marine plants and animals, showing the immense diversity found within this environment. These marine natural products or bioactive compounds may inhibit one or more stages of carcinogenesis by preventing development or treating cancer [1,2]. Among major marine compounds known to display anti-inflammatory and anti-cancer effects, omega 3 polyunsaturated fatty acids (omega 3 PUFAs) are likely the most studied [2,3]. Omega-3 polyunsaturated fatty acids (PUFAs) are essential for human health and are found primarily in coldwater fish in the form of eicosapentaenoic acid (EPA; 20:5n-3) and docosahexaenoic acid (DHA; 22:6n-3) [1,4]. In order to obtain an efficient dose and health beneficial effects, various formulations of omega 3 PUFA supplements are available, such as free fatty acids, ethyl esters, triacylglycerides, phospholipids, and monoacylglycerides.

Studies have shown that omega 3 fatty acids in monoacylglyceride forms did not required pancreatic lipase to be absorbed by the gastro intestinal tract: this property confers increased absorption, and thus a higher bioavailability when compared to other formulations [5,6,7,8]. Moreover, pharmacokinetic analyses clearly demonstrated that MAG-DHA increases the bioavailability of DHA compared to triacylglyceride (DHA-TG) or DHA ethyl ester (DHA-EE) [6,7]. Our previous study has shown a better absorption of omega 3 PUFA monoacylglycerides in cultured human colorectal cancer cells when compared to the absorption rate of the corresponding free PUFA [8]. Colorectal cancer (CRC) is the second most common cause of cancer mortality [9]. Diet factors and nutritional disorders also play important roles in tumorigenesis and tumor development [4]. Despite the emergence of new targeted agents and the use of various therapeutic combinations, none of the treatment options available are curative in patients with advanced CRC [9]. The aim of this study was to determine the anti-cancer effects of eicosapentaenoic acid monoglyceride (MAG-EPA). The specific objective was to evaluate the effect of MAG-EPA on tumor growth and VEGFR and EGFR activation pathways using an in vitro model of human colorectal cancer cell line HCT116 and an in vivo mouse model of HCT116 xenograft.

## 2. Results

### 2.1. Effect of n-3 PUFA Monoglyceride Treatments on Cell Proliferation and Apoptosis in HCT116 Cells

**Figure 1:**
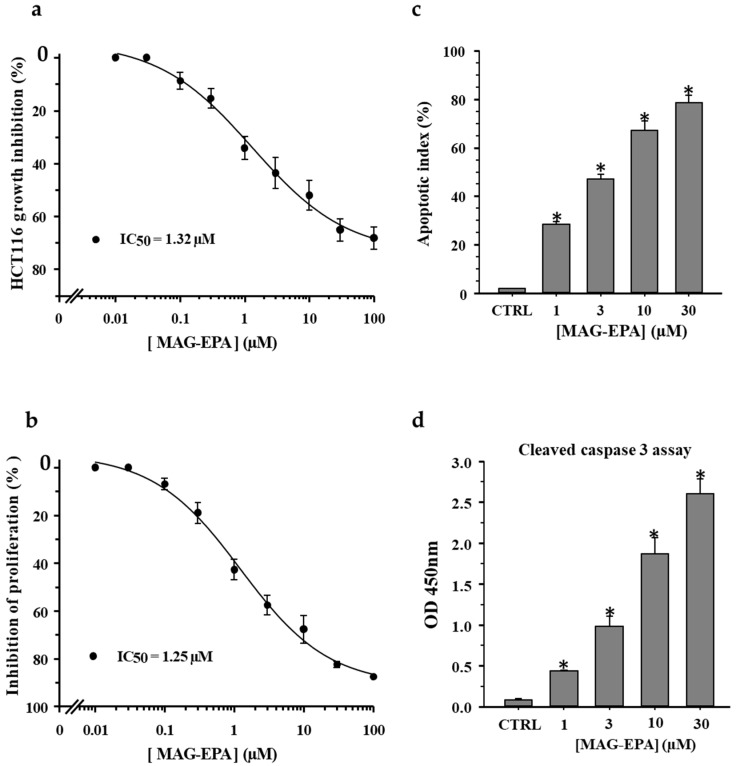
Effect of eicosapentaenoic acid monoglyceride (MAG-EPA) treatments on growth, proliferation and apoptosis levels in HCT116 cells. (**a**) Cumulative concentration response curves (CCRC) displaying the growth inhibitory effect induced by MAG-EPA in HCT116 cells (*n* = 8 for each experimental condition). (**b**) CCRC showing the MAG-EPA inhibitory effect on proliferation of HCT116 following BrdU assay (*n* = 6). (**c**) Bar histogram displaying the apoptotic index calculated following TUNEL assay for HCT116 cells in control and treated with 1 μM, 3 μM, 10 μM and 30 μM MAG-EPA (*n* = 6 for each experimental condition * *p* < 0.05). (**d**) Mean cleaved caspase 3 activity was determined using specific ELISA on cell lysates derived from control and MAG-EPA-treated cells (*n* = 6 * *p* < 0.05).

The effect of MAG-EPA treatments was evaluated on growth inhibition in human HCT116 cells. The cells were treated with increasing concentrations of MAG-EPA (0.01 μM, 0.03 μM, 0.1 μM, 0.3 μM, 1 μM, 3 μM, 10 μM, 30 μM and 100 μM) and counted using a Countess Automated Cell Counter. Cumulative concentration response curves (CCRC) are illustrated on Figure 1a, and results demonstrated that MAG-EPA reduced cell growth of HCT116 cells, with an IC_50_ value of 1.32 ± 0.05 μM (Figure 1a). BrdU assay was performed to evaluate the effect of incremental concentrations of MAG-EPA on cell proliferation rate. CCRC revealed that MAG-EPA significantly reduced the HCT116 cell proliferation, with IC_50_ value of 1.25 ± 0.03 μM (Figure 1b). TUNEL assay was performed to determine the ability of MAG-EPA to induce apoptosis in HCT116 cells. Data revealed that 1 μM, 3 μM, 10 μM and 30 μM MAG-EPA induced apoptosis of HCT116 cells in a concentration-dependent manner (Figure 1c). Moreover, we performed an ELISA assay to evaluate the effect of MAG-EPA on caspase-3 activation. Figure 1d showed that 1 μM, 3 μM, 10 μM and 30 μM of MAG-EPA induced activation of caspase 3 in HCT116 cells (Figure 1d).

### 2.2. Effect of MAG-EPA on HCT116 Spheroids Proliferation and Apoptosis

**Figure 2:**
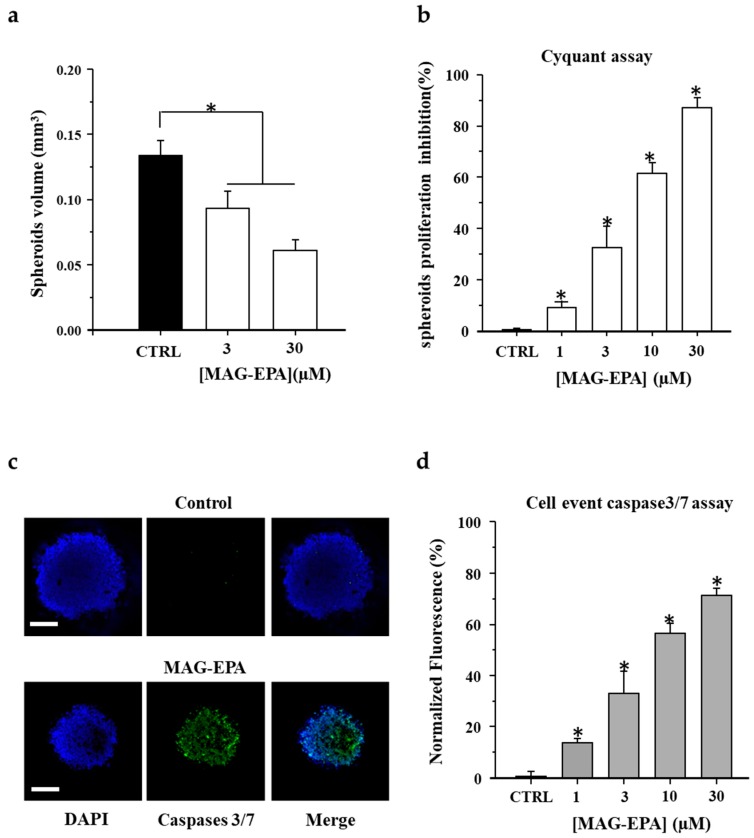
Effect of MAG-EPA on HCT116 cell proliferation and apoptosis in spheroid culture. (**a**) Volume of control and MAG-EPA-treated spheroids, *n* = 15 for each experimental condition, * *p* < 0.05. (**b**) Bar histogram displaying inhibitory effect of MAG-EPA on spheroids proliferation following Cyquant assay, *n* = 12 for each condition. (**c**) Cleaved caspases 3 and 7 signals in HCT116 spheroids in control and following 48 h MAG-EPA treatment (30 μM). Green, caspase 3/7; blue, nuclear DNA (DAPI). Bar 100 μm. (**d**) Mean caspase 3/7 activity was determined using the cell event fluorometric assay on control and MAG-EPA-treated HCT116 spheroids, *n* = 12.

Light microscopy was used to determine the volume of spheroids (4/3·π·*r*^3^) in control and after treatment with MAG-EPA. Results indicate that following 48 h of a 3 μM and 10 μM MAG-EPA treatments, the volumes of HCT116 spheroids were significantly decreased (0.093 ± 0.013 mm^3^ and 0.061 ± 0.008 mm^3^, respectively) when compared to control spheroids (0.134 ± 0.011 mm^3^) (Figure 2a). MAG-EPA treatment was evaluated on spheroids proliferation following Cyquant assay. Quantitative analyses on Figure 2b revealed that 1 μM, 3 μM, 10 μM and 30 μM MAG-EPA decreased the proliferation rate of HCT116 cells in a concentration-dependent manner. Immunofluorescence staining using a specific antibody against cleaved caspases 3 and 7 was performed on HCT116 spheroids derived from control (untreated) and from 30 μM MAG-EPA-treated spheroids. In these spheroids, a 48 h treatment with 30 μM MAG-EPA consistently increased cleaved caspases 3 and 7 staining when compared to control (untreated) HCT116 spheroids (Figure 2c). Quantitative analysis demonstrated a significant increasing in cleaved caspases 3 and 7 in cells derived from 1 μM, 3 μM, 10 μM and 30 μM MAG-EPA-treated spheroids when compared to control (untreated) spheroids (Figure 2d).

### 2.3. MAG-EPA Treatment Reduced HCT116-Induced Tumor Formation

**Figure 3:**
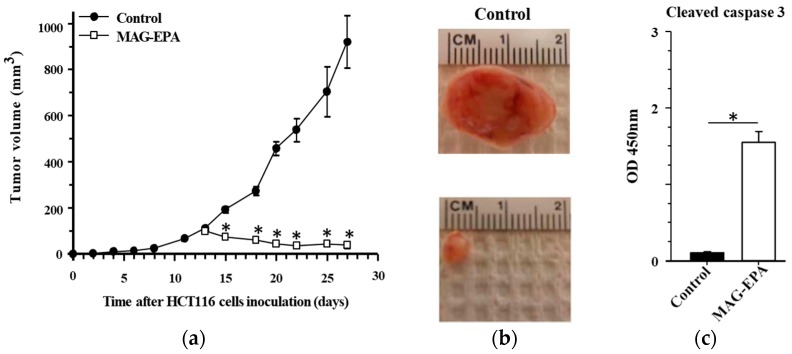
Effect of MAG-EPA treatment on tumor growth in the HCT116 xenograft nude mice model. (**a**) Tumor growth (mm^3^) as a function of time (day) was measured after subcutaneous injection of 1 × 10^6^ HCT116 cells in control and MAG-EPA (618 mg/kg)-treated mice. Results represent the mean volume ± SEM (*n* = 6 per group). (**b**) Representative macroscopic images of tumor size derived from control and MAG-EPA-treated mice. (**c**) Cleaved caspase-3 levels were determined using specific ELISA in tumor homogenates derived from control and MAG-EPA-treated mice, *n* = 6, * *p* < 0.05.

To determine the ability of MAG-EPA to reduce tumor formation an in vivo HCT116 xenograft model was used. Following the formation of 100 mm^3^ tumors, the mice were randomly divided into two groups at day 13, namely control and MAG-EPA-treated. Mice received MAG-EPA *per os* daily at a pharmacological dose of 618 mg/kg corresponding to a daily omega-3 dose of 3 g for humans (Health Canada fish oil monograph), following dose translation of human to mouse by the equation described by Reagan-Shaw [10]. Results revealed that the MAG-EPA treatment was able to arrest tumor growth and reduce the size of the tumor observed prior to treatment (Figure 3a). Moreover, at day 13, the mean tumor volume was 101.7 ± 2.6 mm^3^, whereas after 14 daily treatments, a 3-fold reduction relative to initial tumor size was observed with a mean tumor volume of 38.4 ± 14.2 mm^3^ at day 27. No significant difference in mice weight was found between groups. Representative macroscopic images demonstrated the reduction of tumor size in MAG-EPA-treated mice compared to control animals (Figure 3b). Caspase-3 activation assay was performed in order to identify the effect of MAG-EPA treatment on the level of cleaved caspase-3 detected in tumor homogenates. Results demonstrated that MAG-EPA induced a significant activation of caspase-3 in tumor tissue (Figure 3c) compared with tumor homogenates derived from control mice.

### 2.4. Effect of MAG-EPA on VEGF, HIF1α and VEGFR Phosphorylation Level

**Figure 4:**
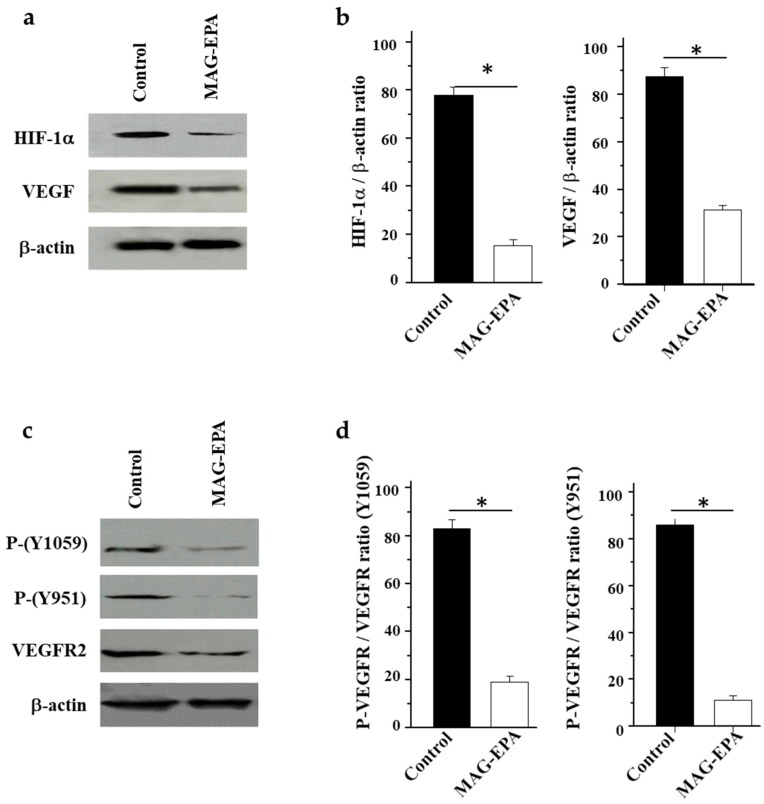
Effect of MAG-EPA on HIF-1α and vascular endothelial growth factor (VEGF) expression and VEGFR2 phosphorylation levels in tumor homogenates. (**a**) Proteins from distinct homogenates were stained using specific antibodies against HIF-1α, VEGF and β-actin. (**b**) Quantitative analyses of mean HIF-1α/β-actin and VEGF/β-actin density ratios, (*n* = 6, * *p* < 0.05). (**c**) Western blot analysis of phosphorylated forms of VEGFR2, P-VEGFR (Y1059) and P-VEGFR (Y951), total VEGFR2 and β-actin protein detection (*n* = 6). (**d**) Quantitative analyses of P-VEGFR2/VEGFR2 mean density ratios, (*n* = 6, * *p* < 0.05).

To determine whether MAG-EPA induced the reduction in the tumor formation and anti-cancer effects, the protein levels of VEGF and HIF1α, as well as the phosphorylation level of VEGFR were determined in tumors derived from control and MAG-EPA-treated animals. Figure 4a,b illustrate the representative western blot and quantitative analyses performed on tumor homogenates derived from control and MAG-EPA-treated mice using antibodies against VEGF, HIF1α, and β-actin. Results revealed that MAG-EPA treatments reduced VEGF and HIF1α expression levels in tumors when compared with the tumors derived from control mice (Figure 4a). Significant reductions of 64 ± 3.1% and 80 ± 4.2% were quantified for expression levels of VEGF and HIF1α in MAG-EPA-treated mice following a comparative analysis of VEGF/β-actin and HIF1α/β-actin ratios after the normalization of identical immunoblot membrane areas (Figure 4b). Western blot analyses using antibodies against total VEGFR and β-actin as well as phosphorylated forms of VEGFR (P-Y1059 and P-Y951) were performed on tumor homogenates derived from control and MAG-EPA-treated mice (Figure 4c). Results revealed that the MAG-EPA treatments decreased the phosphorylation levels of VEGFR in tumors comparatively to those observed in the control group (Figure 4c). Significant reductions of 77% and 87% were quantified for phosphorylated levels of P-Y1059 and Y951 in MAG-EPA-treated mice following comparative analysis of P-VEGFR/total VEGFR ratio following the normalization of identical immunoblot membrane areas (Figure 4d).

### 2.5. MAG-EPA Reduced EGFR and AKT Phosphorylation Levels in Tumor Tissues

**Figure 5:**
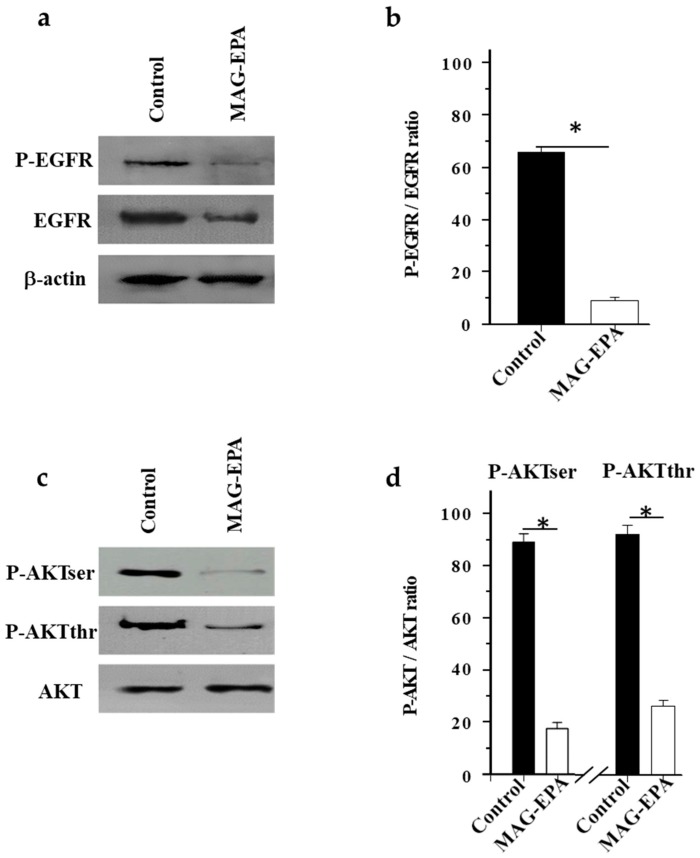
Effect of MAG-EPA on phosphorylation state of EGFR in tumor homogenates. (**a**) Western blot analysis of tumor homogenate protein fractions derived from control and MAG-EPA-treated mice, using specific antibodies against phosphorylated form of EGFR, total EGFR, and β-actin. (**b**) Quantitative analysis of P-EGFR/EGFR density ratios as a function of experimental conditions (*n* = 6, * *p* < 0.05). (**c**) Western blot analysis of phosphorylated forms of AKT (P-AKTser, P-AKTthr) and total AKT protein detection (*n* = 6). (**d**) Quantitative analyses of P-AKT/AKT mean density ratios, (*n* = 6, * *p* < 0.05).

Western blot analyses were performed to assess the expression level of total and phosphorylated forms of EGFR in tumor homogenates derived from the control and MAG-EPA-treated mice. Analyses revealed a reduced EGFR phosphorylation level in tumor homogenates derived from MAG-EPA-treated mice comparatively to control mice (Figure 5a). Moreover, a reduction of total EGFR protein expression was observed following the MAG-EPA treatment. β-actin staining remained constant under the experimental conditions (Figure 5a). Upon quantitative comparative analysis of the P-EGFR/EGFR ratio after normalization of identical immunoblot membrane areas (Figure 5b), results showed that MAG-EPA decreased the EGFR phosphorylation levels by 86% compared to the control group. Further experiments were also performed to evaluate the effect of MAG-EPA on AKT phosphorylation levels. Western blot analysis of tumor homogenates derived from these preparations revealed a reduction in AKT phosphorylation levels following MAG-EPA treatment when compared with control mice (Figure 5c), which translated into significant reductions of 80% and 71% in P-AKTser/AKT and P-AKTthr/AKT ratios, respectively, after the normalization of identical immunoblot membrane areas (Figure 5d).

### 2.6. Comparative Effects of Krill Oil, EPA-EE and MAG-EPA Treatment on HCT116-Induced Tumor Formation

**Figure 6:**
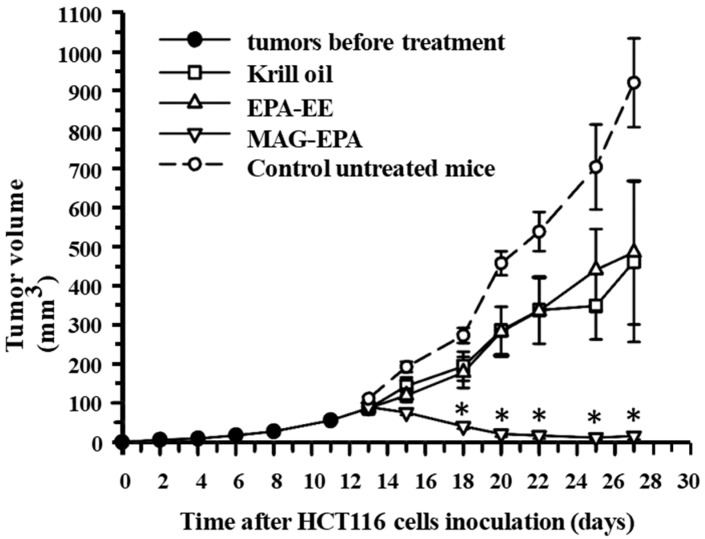
Effect of EPA-EE and Krill oil treatment on tumor growth in the HCT116 xenograft nude mice model. Tumor growth (mm^3^) as a function of time (day) was measured after subcutaneous injection of 1 × 10^6^ HCT116 cells in EPA-EE (618 mg/kg), Krill oil (618 mg/kg) and MAG-EPA (618 mg/kg)-treated mice. Compounds were administered orally daily dose of 618 mg/kg total omega-3 from day 13 to day 27. Results represent the mean volume ± SEM (*n* = 6 per group, * *p* < 0.05).

An in vivo nude mice HCT116 xenograft model was used to compare the ability of MAG-EPA to reduce tumor formation vs Krill oil and EPA-EE. Following the formation of 100 mm^3^ tumors, mice were randomly divided into three groups at day 13, namely Krill oil, EPA-EE, and MAG-EPA-treated. Krill oil, EPA-EE, and MAG-EPA were administered daily *per os* in pharmacological dose (618 mg/kg total omega-3), corresponding to a daily dose of 3.0 g/day total omega-3 for human [10]. Krill oil and EPA-EE treatments slightly reduced tumor latency and growth when compared to control mice- from a previous experiment (Figure 3). No significant reductions of tumor growth were quantified in Krill oil and EPA-EE groups (Figure 6). In contrast, MAG-EPA treatment resulted in a significant reduction of tumor growth when compared to Krill oil or EPA-EE groups (Figure 6). Of note, no significant difference in mice weight was observed between groups. Table 1 illustrates the fatty acids composition of the marine omega-3 oils used for treated mice.

## 3. Discussion

In the present study, MAG-EPA was found to reduce cell proliferation and to induce apoptosis of HCT116 colorectal carcinoma cells. These effects of MAG-EPA in an in-vitro model and an in-vivo mouse xenograft model were correlated with a reduction in phosphorylation levels of VEGFR, EGFR and total VEGF and HIF1α protein expressions as well as an enhanced apoptosis leading to a reduction in tumor cell proliferation.

### 3.1. MAG-EPA Decreases Cell Proliferation and Induces Apoptosis of Colorectal Carcinoma Cells

Previously, we and others showed that PUFAs, especially EPA, DHA, and DPA have an inhibitory effect on the growth of tumor cells both in vitro and in vivo with no cytotoxic action on normal cells [8,11,12,13,14,15]. Tumor cells are known to be deficient in the activity of Δ6 and Δ5 desaturases [16], and thus, may contain substantially lower amounts of EPA and DHA compared to normal cells. Consequently, low amounts of PUFAs in tumor cells may trigger to augment prostaglandins and leukotrienes synthesis as a compensatory mechanism [11]. PGE2 and PGF2α as well as LTA4 and LTB4 are known to be produced in significantly higher amounts by tumor cells, which may enhance their motility and invasive capacity [17,18]. In contrast, PUFAs give rise to potent anti-inflammatory molecules LXs, resolvins, protectins, maresins, and nitrolipids [19,20]. These anti-inflammatory molecules, especially resolvins and protectins have a direct growth inhibitory action on tumor cells [19,21]. Our in vivo results clearly indicate that MAG-EPA was more potent than an equivalent dose of omega-3 in ethyl esters form (EPA-EE) or phospholipids/free fatty acids form (Krill oil) to inhibit tumor growth in mice bearing human colon cancer xenografts. Moreover, when administered orally in a therapeutic mode in mice, MAG-EPA was found to inhibit the growth, and reduced the size of tumors by up to 75% in comparison to tumor size prior to treatment. These observations have clinical relevance since it has been shown that the ratio of dietary ω-6 to ω-3 PUFA is one of the most important factors in determining the cancer risk relative to fatty acid intake [22]. Similarly, another study has demonstrated that the dietary administration of one or both of the main n-3 PUFA in rodent models of colorectal carcinogenesis reduced colorectal tumor size and multiplicity [8,23]. In a population-based prospective study on the association of omega-3 PUFA and cancer, there was an inverse relationship between marine omega-3 PUFA intake and the risk of colorectal cancer [24]. The excellent tolerability and safety profile of omega 3 PUFAs combined with other health benefits, particularly cardiovascular, make MAG-EPA an attractive candidate for prevention and treatment of CRC.

### 3.2. MAG-EPA Decreases VEGFR and EGFR Activation Pathways in Colorectal Carcinoma Cells

In an attempt to understand the mechanism by which MAG-EPA mediates its antitumor effects in colorectal cancer cells, we determined whether this monoglyceride form of omega-3 modulates VEGFR and EGFR signaling pathways in tumor homogenates derived from control (untreated) and MAG-EPA-treated mice. Vascular endothelial growth factor (VEGF) is a potent angiogenic factor associated with tumor progression and metastasis in numerous solid malignancies [25]. VEGF binds to three tyrosine kinase receptors: VEGFR-1, VEGFR-2, and VEGFR-3 [25]. Studies have shown VEGFR-2 to be expressed on tumor cells and to be implicated in tumor growth and progression, including activation of the Erk-1/2 and AKT and MAPK pathways [26,27,28]. Moreover, studies have shown that antiangiogenic therapy targeting the tyrosine kinase receptor for the VEGF receptor inhibits the vascularity, proliferation, and growth of colon cancer liver metastasis and significantly increases endothelial and tumor cell apoptosis [25]. In the xenograft model herein, we found that MAG-EPA downregulated the phosphorylated form of VEGFR in tumor when compared to control mice. Moreover, MAG-EPA treatment also slightly reduced VEGFR expression in HCT116 tumors.

The epidermal growth factor receptor (EGFR) is recognized as an important player in colorectal cancer (CRC) initiation and progression [29]. Overexpression of EGFR is common in many tumors. Specifically in CRC, EGFR it is estimated to be overexpressed in 60–80% of tumors, and is associated with a poor prognosis [29]. For these reasons EGFR has been targeted for treatment with small molecule inhibitors and monoclonal antibodies. EGFR is a multifunctional receptor that plays a key role in cell division and apoptosis, cell differentiation and dedifferentiation, and migration [30]. EGFR executes these functions by the activation of multiple signaling pathways including PLC-gamma-1, MAPKs, AKT, JNK and the signal transducers and activators of transcription [30]. HCT116 cells express high levels of EGFR and are a good model to study EGFR modulation by MAG-EPA. We found that the phosphorylated EGFR levels are reduced after treatment with MAG-EPA in tumor derived from HCT116 xenograft mice model when compared to the tumors derived from untreated control animals. Moreover, MAG-EPA treatment also slightly reduced EGFR concentration in HCT116 tumors. Similarly, a study has shown a reduction of EGFR activation in EPA- or DHA-induced apoptosis in breast cancer (MDA-MB-231 and MCF-7) cells, associated to a reduction of Bcl2 and caspase-8 expression [31]. The entire mechanism by which MAG-EPA exert their beneficial effects is not fully understood. However, we have hypothesized that the induction of apoptosis and the reduction of HCT116 cell proliferation induced by MAG-EPA might be explained by a reduced activation of the VEGFR and EGFR pathways, which is correlated with a downregulation of AKT signaling in tumor homogenates. Moreover, MAG-EPA treatment activated the caspase-3, which resulted in an increased number of apoptotic cells in tumor tissues, in agreement with previous studies in which supplementation with fish oil [32] or EPA/DHA [33] significantly inhibited the growth of xenografted tumors in mouse models at a high dose (equivalent to 30 g/day in humans). Further experiments are needed to elucidate whether MAG-EPA induce apoptosis by targeting intrinsic and/or extrinsic pathway.

## 4. Conclusions

In the present study, MAG-EPA may promote apoptosis and inhibit growth of tumors by suppressing EGFR and VEGFR activation pathways, as well as the production of VEGF and HIF1α. Moreover, we and others have recently demonstrated that the anti-proliferative and pro-apoptotic actions of the MAG-EPA compound may partially be explained by the inhibition of signaling pathways including NF-κB, EGFR, VEGFR, and AKT, and the down regulation of its gene products. In consequence, we and others suggests that a balance between pro-inflammatory molecules (PGs, LTs, and TXs) and anti-inflammatory PUFAs-derived molecules (resolvins, protectins) may determine the degree of proliferation and invasive capacity of tumor cells [8,19]. With our current knowledge, we are not able to predict specific PUFA potency, and for future clinical applications, a screening of several PUFA directly on tumor tissue or on isolated circulating tumor cells (CTCs) could be the only way to find the best PUFA for specific cancers.

## 5. Materials and Methods

### 5.1. Marine Omega-3 Oils

MAG-EPA (Solutex, Madrid, Spain) is a fish oil concentrate in monoglycerides form containing 819 mg/g EPA and 39 mg/g DHA. EPA-EE (Equateq Ltd., Isle of Lewis, UK) is a fish oil concentrate in ethyl esters form containing 724 mg/g EPA and 104 mg/g DHA. Krill oil (Jedwards International Inc., Braintree, MA, USA) is an oil in phospholipids and free fatty acids form containing 150 mg/g EPA and 90 mg/g DHA.

### 5.2. Cell Line and Culture

Human HCT116 colorectal adenocarcinoma cells were obtained from the American Type Culture Collection (ATCC). HCT116 cells were maintained in McCoy’s 5A medium (Wisent, St-Bruno, QC, Canada) containing 10% FBS, 5 mM HEPES, and 10 units/mL penicillin, 100 μg/mL streptomycin. Cells were grown in a 5% CO_2_ incubator at 37 °C. Cells were used between passage 3 to 6 for all conditions and assays tested in this study. Cells were untreated or treated with MAG-EPA at the indicated concentrations (0.03–100 μM). MAG-EPA was dissolved in 100% ethanol and diluted to a final concentration of 0.1% ethanol. Cell proliferation analyses were performed using the BrdU cell proliferation assay kit according to the manufacturer’s instructions (New England BioLabs, Pickering, ON, Canada). This kit detects the level of 5-bromo-2’-deoxyuridine (BrdU) incorporated into cellular DNA during cell proliferation using an anti-BrdU antibody. Apoptosis analyses were performed using (1) a terminal deoxynucleotidyl transferase mediated dUTP nick end labeling (TUNEL) assay in accordance with the manufacturer’s instructions (EMD Millipore, Bellerica, MA, USA) and (2) a cleaved caspase-3 ELISA assay according to the manufacturer’s instructions (New England BioLabs, Pickering, ON, Canada) [8].

### 5.3. Spheroids

Human HCT116 colorectal adenocarcinoma cells were cultured in Perfecta3D 96-well hanging drop plate, according to the manufacturer’s instructions (3D Biomatrix Inc., Ann Arbor, MI, USA). Hanging drops were formed using a cell suspension of 5000 cells/well in McCoy’s 5A medium (Wisent, St-Bruno, QC, Canada) containing 10% FBS and 5 mM HEPES. Spheroids were grown in a 5% CO_2_ incubator at 37 °C during 3 weeks. Every day for each well, 10 μL of media was removed and replaced by 14 μL of fresh media. Spheroids were treated directly in wells with final concentrations of MAG-EPA of 1 μM, 3 μM, 10 μM, and 30 μM. Cells proliferation analyses in spheroids were performed using Cyquant assay kit according to the manufacturer’s instructions (Life technologies, Eugene, OR, USA). Immunofluorescence and apoptosis analyses on HCT116 spheroids were performed using Cell event caspase 3/7 assay kit according to the manufacturer’s instructions (Life technologies, Eugene, OR, USA). Image analyses were performed for immunopositive pixel staining of cleaved caspase 3/7 in spheroids sections using an inverted epifluorescence microscope (Nikon-Canada, Mississauga, ON, Canada) and MetaMorph 7.6 software (Molecular Devices, Sunnyvale, CA, USA). DAPI was used as counterstaining.

### 5.4. In Vivo Tumor Xenograft Experiments

Female nu/nu nude mice were obtained from Charles River Laboratories (Montreal, QC, Canada). All studies involving mice were approved by the institutional Animal Care Committee (Protocol: # 237-10). Human HCT116 xenografts were established in 4-week-old nude mice. Mice were subcutaneously inoculated with 0.2 mL of 1 × 10^6^ HCT116 cells in McCoy’s 5A on the right flank. After the formation of 100 mm^3^ tumors, mice were randomly assigned into 4 groups, control (untreated), MAG-EPA-treated (*n* = 8 per group), Krill-Oil-treated, and EPA-EE (ethyl ester)-treated. MAG-EPA was administered *per os* (618 mg/kg) daily. Krill oil and EPA-EE were administered *per os* at a human equivalent of 3.0 g/day of total omega-3. Tumor volumes (V) were calculated using the formula: *V* = (a × b^2^)/2, where “a” is the largest superficial diameter and “b” the smallest. Cleaved caspase-3 ELISA assay was performed on tumor homogenate derived from the control and treated mice according to the manufacturer’s instructions (New England BioLabs, Pickering, ON, Canada). Western blot analyses using specific antibodies against phosphorylated forms of VEGFR (Y1059 and Y951), EGFR, and AKT (at serine and threonine residues) as well as total form of VEGFR, EGFR, AKT, β-actin, VEGF and HIF1α were performed on tumor homogenates derived from control and MAG-EPA-treated mice [8,15].

### 5.5. Data Analysis and Statistics

Results are expressed as means ± S.E.M., with n indicating the number of experiments. Statistical analyses were performed using Sigma Plot 11 and SPSS 14.0 (SPSS-Science, Chicago, IL, USA) via one-way ANOVA followed by Dunnett’s post-hoc test. Differences were considered statistically significant * when *p* < 0.05.

## Figures and Tables

**Table 1:** Fatty acids composition of the marine omega-3 oils. DHA, EPA, and total omega-3 contents were reported in mg/g for Krill oil, EPA-EE, and MAG-EPA.

3.0 g/Day Total Omega-3 Human Equivalent	EPA (mg/g)	DHA (mg/g)	Total Omega-3 (mg/g)
MAG-EPA	819	39	888
EPA-EE	724	104	930
Krill Oil (3X volume)	150	90	300
